# Optimization and Performance Analysis of CAT Method for DNA Sequence Similarity Searching and Alignment

**DOI:** 10.3390/genes15030341

**Published:** 2024-03-07

**Authors:** Veska Gancheva, Hristo Stoev

**Affiliations:** Department of Programming and Computer Technologies, Faculty of Computer Systems and Technologies, Technical University of Sofia, 1756 Sofia, Bulgaria; hristomihaylovstoev@gmail.com

**Keywords:** bioinformatics, biological data, DNA sequences, metadata, performance analysis, similarity searching, sequence alignment

## Abstract

Bioinformatics is a rapidly developing field enabling scientific experiments via computer models and simulations. In recent years, there has been an extraordinary growth in biological databases. Therefore, it is extremely important to propose effective methods and algorithms for the fast and accurate processing of biological data. Sequence comparisons are the best way to investigate and understand the biological functions and evolutionary relationships between genes on the basis of the alignment of two or more DNA sequences in order to maximize the identity level and degree of similarity. This paper presents a new version of the pairwise DNA sequences alignment algorithm, based on a new method called CAT, where a dependency with a previous match and the closest neighbor are taken into consideration to increase the uniqueness of the CAT profile and to reduce possible collisions, i.e., two or more sequence with the same CAT profiles. This makes the proposed algorithm suitable for finding the exact match of a concrete DNA sequence in a large set of DNA data faster. In order to enable the usage of the profiles as sequence metadata, CAT profiles are generated once prior to data uploading to the database. The proposed algorithm consists of two main stages: CAT profile calculation depending on the chosen benchmark sequences and sequence comparison by using the calculated CAT profiles. Improvements in the generation of the CAT profiles are detailed and described in this paper. Block schemes, pseudo code tables, and figures were updated according to the proposed new version and experimental results. Experiments were carried out using the new version of the CAT method for DNA sequence alignment and different datasets. New experimental results regarding collisions, speed, and efficiency of the suggested new implementation are presented. Experiments related to the performance comparison with Needleman–Wunsch were re-executed with the new version of the algorithm to confirm that we have the same performance. A performance analysis of the proposed algorithm based on the CAT method against the Knuth–Morris–Pratt algorithm, which has a complexity of O(n) and is widely used for biological data searching, was performed. The impact of prior matching dependencies on uniqueness for generated CAT profiles is investigated. The experimental results from sequence alignment demonstrate that the proposed CAT method-based algorithm exhibits minimal deviation, which can be deemed negligible if such deviation is considered permissible in favor of enhanced performance. It should be noted that the performance of the CAT algorithm in terms of execution time remains stable, unaffected by the length of the analyzed sequences. Hence, the primary benefit of the suggested approach lies in its rapid processing capabilities in large-scale sequence alignment, a task that traditional exact algorithms would require significantly more time to perform.

## 1. Introduction

The development of technologies for generating biological data—sequencers that generate genetic data—leads to the accumulation of a large volume of data. This surge in bioinformatics data is propelled by the swift advancements in high-throughput sequencing projects. Every day, an extraordinary volume of data is produced, encompassing clinical reports, genomic sequences, gene expression profiles, biomedical literature reviews, medical imagery, and sensor outputs. For instance, the European Bioinformatics Institute is reported to hold around 390 petabytes of raw data storage, which includes information on genes, small molecules, and proteins [1]. This rapid data growth has spurred the development and study of numerous solutions for analyzing biological data, such as genome-wide association studies, sequence alignment, single nucleotide polymorphism detection, and genome assembly. These applications often share several characteristics: (1) vast volume of data generated in sequencing centers; (2) lengthy processing times, exemplified by the genome assembly tool SOAPdenovo [2], which can take days and consume hundreds of GB of memory to finish construction of a single human genome; and (3) application dependency. Achieving the results, from which valuable insights can be derived, requires undergoing various processing stages, thereby incurring significant operational costs due to data transmission.

In biology, it is necessary to understand how similar two sequences are to each other, for example, sequences of amino acids making up a protein molecule or sequences of nucleic acids in a DNA molecule. In bioinformatics, a computer model is built of the DNA molecule, which is represented as a string of 4-letter alphabets, and of the protein molecule, which is represented as a string of 20-letter alphabets. The nucleotide or protein sequences being compared are commonly presented as rows in the matrix. Between residues are inserted gaps, so identical characters are arranged in consecutive columns.

Sequence comparisons are the best way to investigate evolutionary relationships between genes on the basis of the alignment of two or more DNA sequences in order to maximize the identity level and degree of similarity possibility of homology. Sequence alignment algorithms are used to compare and search DNA or protein databases. They have emerged as one of the strongest techniques to assist in identifying the biological functions of a given gene. The mainstream of searchable data in such a database is a nucleotide or protein sequence. Hence, in order to obtain information about a new biological sequence, one of the first steps is a comparison with a group of already known ones from the database. Frequently, the outcomes imply functional, structural, or evolutionary similarities among the sequences.

A critical aspect of biological data processing involves identifying homologous sequences in databases. Although algorithms like Needleman–Wunsch [3], Smith–Waterman [4], and Knuth–Morris–Pratt [5] accurately measure similarities between two sequences, applying them to large datasets is time-consuming. To expedite searches in substantial databases, researchers employ heuristic methods and algorithms, which, while accelerating search times, may compromise result quality. The FASTA version 36 software package, designed for aligning DNA and protein sequences, incorporates heuristic approaches for querying entire databases. BLAST, a widely used sequence search tool [6,7], employs a faster heuristic algorithm than optimal alignment approaches, enhancing search efficiency without sacrificing sensitivity. 

A metaheuristic method for multiple sequence alignment involves generating a favorite sequence, serving as a benchmark for comparing all sequences within the database [8]. Challenges arise in applying this approach when introducing new data or removing existing records:

1. When data change, the favorite sequence requires recalculation.
2. Re-comparing each database sequence with the updated favorite sequence requires the expenditure of computing time and resources.
3. Each database has a distinct favorite sequence, complicating the merging of databases, particularly in extensive datasets with diverse structures and access methods.

### 1.1. Proposed Research Objectives

In seeking to enhance existing heuristic algorithms, our research aims to introduce improvements in three key areas.

#### 1.1.1. Constant Favorite Sequence

We aim to devise a method for establishing a constant favorite sequence independent of the data in the database, ensuring that it remains unchanged even if the database undergoes modifications. This approach seeks to provide stability in sequence alignment, mitigating the need for frequent recalculations of the favorite sequence.

#### 1.1.2. Minimizing Comparisons with Favorite Sequence

Our research endeavors to minimize the comparisons with the favorite sequence during database search. Typically, each sequence involves a complex comparison algorithm against the favorite sequence. By optimizing this process, we intend to enhance the efficiency of the alignment process, making it more resource-effective.

#### 1.1.3. Unification of Sequence Favorites across Databases

To address challenges in database merging, we propose a unified approach to sequence favorites. This involves developing a method to unify sequence favorites for all databases, ensuring compatibility and coherence in large-scale data scenarios. This unification aims to streamline the management and analysis of biological data across diverse databases.

### 1.2. Research Purpose and Methodology

The overarching aim of this investigation is to introduce a novel pairwise DNA sequence alignment algorithm. Our approach leverages a novel, effective, and comprehensive DNA sequence alignment method, employing trilateration. The primary goal is to offer solutions to three fundamental sequence alignment issues:

(1) Constant Favorite Sequence: introduce a methodology to create a stable and constant favorite sequence, reducing the need for frequent recalculations.

(2) Reduced Comparisons with Favorite Sequence: develop strategies to minimize the frequency of comparisons with the favorite sequence throughout the alignment process, enhancing computational efficiency.

(3) Unified Favorite Sequences: propose a method to unify favorite sequences across all databases, providing a standardized approach to sequence alignment in diverse data environments.

Via this comprehensive approach, we aim to contribute to the advancement of bioinformatics methodologies, addressing key challenges in DNA sequence alignment. The research included in this article is an extension of the research presented in [9]. 

This paper introduces an enhanced version of the CAT algorithm for pairwise DNA sequence alignment, incorporating novel elements that consider dependencies on previous matches and proximity to the closest neighbor. These adjustments aim to augment the distinctiveness of the CAT profiles and minimize potential collisions where two or more sequences share identical CAT profiles. Such advancements render the revised algorithm more efficient for accurately identifying exact matches within extensive DNA datasets quickly. The improvements made to the CAT profile generation process are elaborated. Changes occur in the first stage of the calculation of the CAT profiles, and they do not affect the second stage, which is the actual comparison.

New results from the same experiments are presented. With the new version, all experiments related to the examination of collisions were re-executed; no collisions were found, and some of the figures in the original article became redundant and were removed. Block schemes, pseudocode, tables, and figures are updated according to the proposed new version and experimental results. Additionally, this paper presents experimental results derived from applying this updated version of the CAT method across various datasets for DNA sequence alignment.

The following sections of the research will delve into the specific methodologies, results, and conclusions related to each of these proposed improvements.

## 2. Materials and Methods

### 2.1. Methods and Algorithms for Sequence Alignment

String alignment algorithms are widely used in bioinformatics to compare DNA sequences. The pattern of a particular DNA sequence in the form of a character string is compared or searched against other sequences to find similarities [10]. Therefore, to match a pattern against a vast array of DNA sequences, which can be intricate and challenging to analyze, algorithms like Knuth–Morris–Pratt, Boyer–Moore, Brute Force, and Rabin–Karp [11], among others, are employed to achieve more accurate results or pattern matches. 

A number of studies have been conducted on string-matching algorithms, including exact matching and approximate string matching [12,13]. The complexity of these algorithms is evaluated using DNA datasets to identify the most efficient algorithm that balances speed and accuracy effectively. Global alignment, as a strategy for global optimization, involves aligning sequences in their entirety, covering the full length of the sequences under analysis. Local alignment identifies similar areas in long sequences that are generally too different.

In the field of bioinformatics, two primary types of algorithms to align pairs of sequences are used: exact and approximate (heuristic-based) [14]. Exact algorithms utilize dynamic programming techniques to ensure that the optimal alignment between sequences is found, considering every possible alignment, and scoring them based on a predefined scoring system [15,16]. This approach guarantees the identification of the best possible alignment but can be computationally intensive, especially for long sequences. Examples include Smith–Waterman and Needleman–Wunsch algorithms, which tend to be very computationally complex but manage to find the optimal arrangement between pairs of sequences. FASTA [17,18] and BLAST are heuristic-based algorithms that are more widely used because they offer faster computational performance. The challenge in performing sequence alignment from biological data is the trade-off between accuracy and efficiency. 

The Needleman–Wunsch algorithm is designed for global alignment, which means that it aligns two sequences from beginning to end, optimizing for the best possible match across the entire length of both sequences. Because of this, it is not tailored to identify local regions of similarity within larger sequences where only a portion might be similar. This algorithm tries to reach the maximum of matches and the minimum of mismatches (gaps) when comparing protein or nucleotide sequences. The Needleman–Wunsch algorithm, which employs dynamic programming, ensures the computation of the maximum score for aligning two sequences. This method systematically examines all possible alignments between the sequences, calculating and comparing their scores to identify the alignment with the highest possible score.

The Smith–Waterman algorithm implements local sequence alignment, pinpointing regions of highest similarity rather than aligning sequences in their entirety. It is designed to identify similar regions between strings of nucleotides or proteins, making it especially useful for analyzing sequences where the most conserved and functionally relevant sections may not align from end to end. Often, the terminal regions of proteins exhibit higher variability due to increased rates of mutations, deletions, and insertions, rendering the middle sections more critical for comparative analysis. Based on dynamic programming, the Smith–Waterman algorithm calculates the optimal local alignment score by considering all possible segment pairings between two sequences, ensuring high precision in identifying regions of similarity. Its computational complexity for aligning two sequences is O(MN), with M and N representing the lengths of these sequences. However, given the rapid expansion of genetic databases, the practical computational demand effectively scales to O(kMN), where k indicates the growth rate of databases. This emphasizes the algorithm’s intensive computational requirements in the context of the burgeoning volume of genetic sequence data.

The first developed and implemented popular heuristic algorithm for database similarity search is FASTA, which can be used for the rapid comparison of protein or nucleotide sequences. The FASTA algorithm is most generally used for biological sequence database searching. The short identical sections in the two sequences can be located. A sequence of multiple matching regions that are observed in the same order in both sequences is used as the starting point for dynamic programming algorithm ordering. This algorithm attains a significant degree of similarity search accuracy and rapid performance due to the use of a word-matching model. Its objective is to identify a likely match prior to initiating the detailed, optimized search process. The balance between speed and precision is regulated by a parameter that sets the word size. Rather than seeking matches for every word, the FASTA algorithm looks for segments that include multiple consecutive matches. These segments are compared using a heuristic method to determine the segment with the best score.

The BLAST algorithm is perhaps the most widely used tool that has been developed for bioinformatics research purposes. It is also heuristic-based and is used to search for homologous sequences. Sequences that have k-fold matches are found in the database using the BLAST algorithm. BLAST creates a look-up table of all substrings of the given input length contained in the input sequence, as well as similar “neighboring” substrings. It is used to compare sequences with biological information, both for sequences containing amino acids of different proteins and for sequences containing nucleotides of DNA. The BLAST algorithm compares a given sequence to a database of sequences.

Different variants of the BLAST algorithm have been designed and implemented (MEGABLAST and PSIBLAST). As the biological database volume expands, it is imperative to compare, search, and analyze the data using parallel algorithms and high-performance computers. Several parallel versions of the BLAST alignment and search algorithm have been developed. mpiBLAST uses the database segmentation strategy. It can be used on different types of computer clusters and supercomputers. It is very popular among bioinformatics scientists in need of a high-throughput BLAST algorithm. Many scientists have reported experimental results of their research works using parallel implementations of the BLAST package [19,20,21,22,23].

However, even parallel execution of sequence alignment algorithms faces limitations on hardware systems [24,25,26]. A novel approach for sequence comparison is proposed by defining a heuristic alignment within the database environment [27]. This approach leverages the strengths of the database management system, offering a strategy to utilize similarities within datasets to expedite the alignment process. Deep learning models are also used to predict miRNA binding sites [28]. A technique known as imputation sequence alignment has been introduced for miRNA target site prediction models. This method enables the interpretation of deep learning models by using a two-dimensional representation of miRNA and potential target sequences. A different strategy for identifying DNA sequence similarities involves establishing a generalized string editing distance. This approach permits not only single nucleotide modifications but also the insertion or deletion of complete motifs. A dynamic programming approach has been crafted to calculate this distance between sequences [29].

### 2.2. CAT Method for DNA Sequence Alignment Based on Trilateration

Finding a beginning point, or benchmark, against which the other data in the database can be compared is at the core of the concept of a favorite sequence. Alternatively, if we were to approach the problem mathematically, sequence favorite could be represented as a function of N unknowns (in the context of DNA, the unknowns are the 4 bases adenine, thymine, guanine, and cytosine), and the remaining database entries could then be represented once more as functions of the same variables. In this scenario, the distance between each individual sequence and the preferred sequence would be represented by the similarity comparison. In other words, determine the relationship between a point defined by the favorite sequence function and a point described by the sequence function. 

A point is generated somewhere in the center of the cloud of points that is used as a reference (sequence favorite) when compared to a set of points (the database entries) because there is no coordinate system. However, if a coordinate system or three or more reference points are found, it would be possible to use trilateration or elementary analytical geometry to determine the positions of the points relative to one another, which would reflect the degree to which the database records match one another. Moreover, to it would eliminate the requirement for favorite computation sequences.

A novel approach, known as the CAT method (named after the first letter of the defined constant benchmarks C, A, and T), has been introduced for aligning DNA sequences, utilizing the trilateration technique [30]. This method establishes three consistent reference points for trilateration application, resulting in a steadfast reference sequence. This sequence, comprising the C-benchmark, A-benchmark, and T-benchmark, remains constant regardless of alterations in the database content. 

Key points establishing the reference benchmark are as follows:

- For the A-benchmark and T-benchmark, we should never have a matching position and base. In this way, the base from processed DNA could match on index and base on A or T, but not both.
- For the C-benchmark, we want an index and base of 25% of the A-benchmark and 25% of the T-benchmark to match the C-benchmark.

ACGTACGTACGTACGTACGTACGTACGTACGTACGTAC……—A-Benchmark

GTACGTACGTACGTACGTACGTACGTACGTACGTACGT……—T-Benchmark

AGTCAGTCAGTCAGTCAGTCAGTCAGTCAGTCAGTCAG……—C-Benchmark

With the establishment of the three benchmark conversions for the needs of the trilateration method, concern (1) is no longer an issue. Constant sequence favorite means that changes in the dataset would not lead to further recalculation anymore.

Liberation of the benchmark conventions from the dataset records allows for time-consuming calculations to be made during the upload processes of the data into the database and for CAT profiles to accompany information for the sequence. This is how, during the look up, heavy calculations are omitted, and instead, the CAT profiles are used for the actual comparison. 

The establishment of benchmark conversions gives another advantage—unification of favorite sequences for all databases—elimination of concern (3). With the unified sequences that are standardized for all databases based on the described alignment algorithm, when two sequences have same profiles, one hundred percent complete matching of one sequence on the other can be expected.

**Figure 1:**
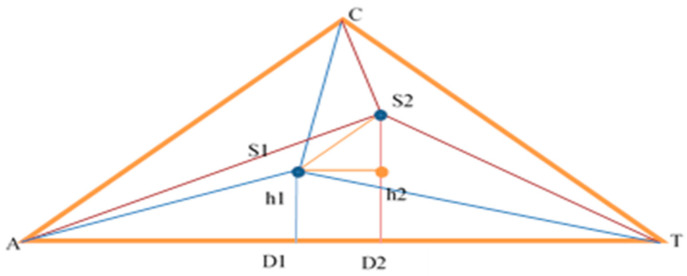
Calculation of the distance between two profiles.

For the evaluation of two sequences, it is necessary to calculate the distance of the segment *S*_1_*S*_2_ in Figure 1.

(1) $$S_{1}S_{2}=\sqrt{{\left|{AD}_{1}-{AD}_{2}\right|}^{2\;}+{\left|h_{1}-h_{2}\right|}^{2\;}}$$

For now, ∆*AS*_1_*T* is considered, and then analogous calculations and reasoning are performed for *AS*_2_*T*. What is known about ∆*AS*_1_*T* is the sides *AT* = |1|, *AS*_1_ = distance from *S*_1_ to A-benchmark (it is known), and *S*_1_*T* = distance from *S*_1_ to the T-benchmark. The cosine theorem is used to find ∡*TAC* and then side *AD*_1_:

(2) $${S_{1}T}^{2}={{AS}_{1}}^{2}+{AT}^{2}-2{\cdot AS}_{1}\cdot AT\cdot \cos\left(\alpha_{1}\right)$$

(3) $$\cos\left(\alpha_{1}\right)=\frac{{{AS}_{1}}^{2}+{AT}^{2}-{S_{1}T}^{2}}{2\cdot AS_{1}\cdot AT}$$

(4) $${AD}_{1}=AS_{1}\cdot \cos\left(\alpha_{1}\right)$$

(5) $$h_{1}=\sqrt{AS_{1}^{2}-({AS_{1}\cdot cos(\alpha_{1}))}^{2}}$$

The calculations for triangle AS_2_T are analogous. After substituting the values found, a value for *S*_1_*S*_2_ is obtained.

(6) $$S_{1}S_{2}=\sqrt{{\left|{AD}_{1}-{AD}_{2}\right|}^{2\;}+{\left|h_{1}-h_{2}\right|}^{2\;}}$$

The smaller value obtained for the intercept, the greater the probability of a complete match, expressed as a percentage. This simple Formula (6) gives us the freedom to iterate over the database records quickly and to search for results with a certain percentage of similarity, which can be aligned and compared further with more accurate algorithms such as Needleman–Wunsch or Smith–Waterman.

It is possible for collisions to occur in such a proposed DNA sequence alignment method based on trilateration, i.e.,

1. More than one sequence of the same length to obtain the same values for *AD* and *h*:
  - Due to the nature of benchmark sequences and the fact that the real sequence projects at most a quarter of the bases onto the benchmark, i.e., with benchmark ACGT and projection of G at the second position, there are no matches and no value is accumulated for the match rate.
2. During *S*_1_*S*_2_ calculation, the same values are obtained:
  - Because of statistical errors accumulated when calculating *AD* and *h*.
  - Because of rounding in calculations due to the range of data types, this cannot be avoided even with the use of more precise types.

To minimize collisions, the precision in calculations for *AD* and *h* should be increased by adding the dependency on neighboring bases. Like in local alignment, when the current base of the benchmark sequence does not match the current base of the real sequence, additional points can be added or subtracted, depending on whether a neighboring base match is. After Needleman–Wunsch alignment, the places where the bases do not match appear as gap “_” positions and are given different points accordingly. 

A similar principle can be applied to the proposed method if a base and index match is given a value of 1. If there is a mismatch, a neighboring base from the benchmark sequence is checked and given a value of 0.6 or 0.4 depending on how far the neighbor is, i.e., when there is match with the left or right base from the benchmark, it is given 0.6; when it is far, it is given 0.4. If we define the baseDistance array for near matches, it will look like [0, 0.6, 0.4, 0.6]. Left and right neighbors for indexes 1 and 3; the 0 index is for exact matches; and index 2 is the far neighbor.

Example of benchmark ACGT base at position 2 G:

ACGT

XGXX

G at position 2 corresponds to C from the benchmark sequence, and instead of 0, a match value of 0.6 can be given because it is adjacent to the right; in Needleman–Wunsch ordering, it has the following alignment:

ACG_T

X_GXX

In this way, the precision of AD and h calculation is increased, and the accumulation of statistical errors is reduced 1. thus reducing collisions and 2. after finding a suitable sequence in the base, one with a minimum value for *S*_1_*S*_2_, a more accurate alignment algorithm can be applied. The comparison calculations in the direction calculate the CAT of two sections proposed in the presented method with a constant complexity that makes it applicable as a first step suitable for the FASTA algorithm as well as for multiple alignments such as ClustalW.

Dependency with a previous match can be added to the current position to increase the uniqueness of the CAT profile. The positional numeral system contribution of a digit to the value of a number is the value of the digit multiplied by a factor determined by the position of the digit. Something like this needs to be carried out here, but considering the average length of the sequence, the exact same approach cannot be used. Instead, when a previous exact or near match is detected, a sum of the current maximum theoretical sequence of matches is tracked and will be increased. The ratio of a current maximum theoretical sequence of matches to the current index will be added to the sum of previous matches, and this will be considered as a kind of bonus point. All given bonuses from previous matches must be tracked as well since they are needed in a later stage to correct the distances so that a triangle can be formed. To apply the method of trilateration, calculated distances should ensure that in any conditions, a triangle can be formed, i.e., the sum of any two sides must be greater than the third one.

### 2.3. DNA Sequence Alignment Algorithm Based on CAT Method

**Figure 2:**
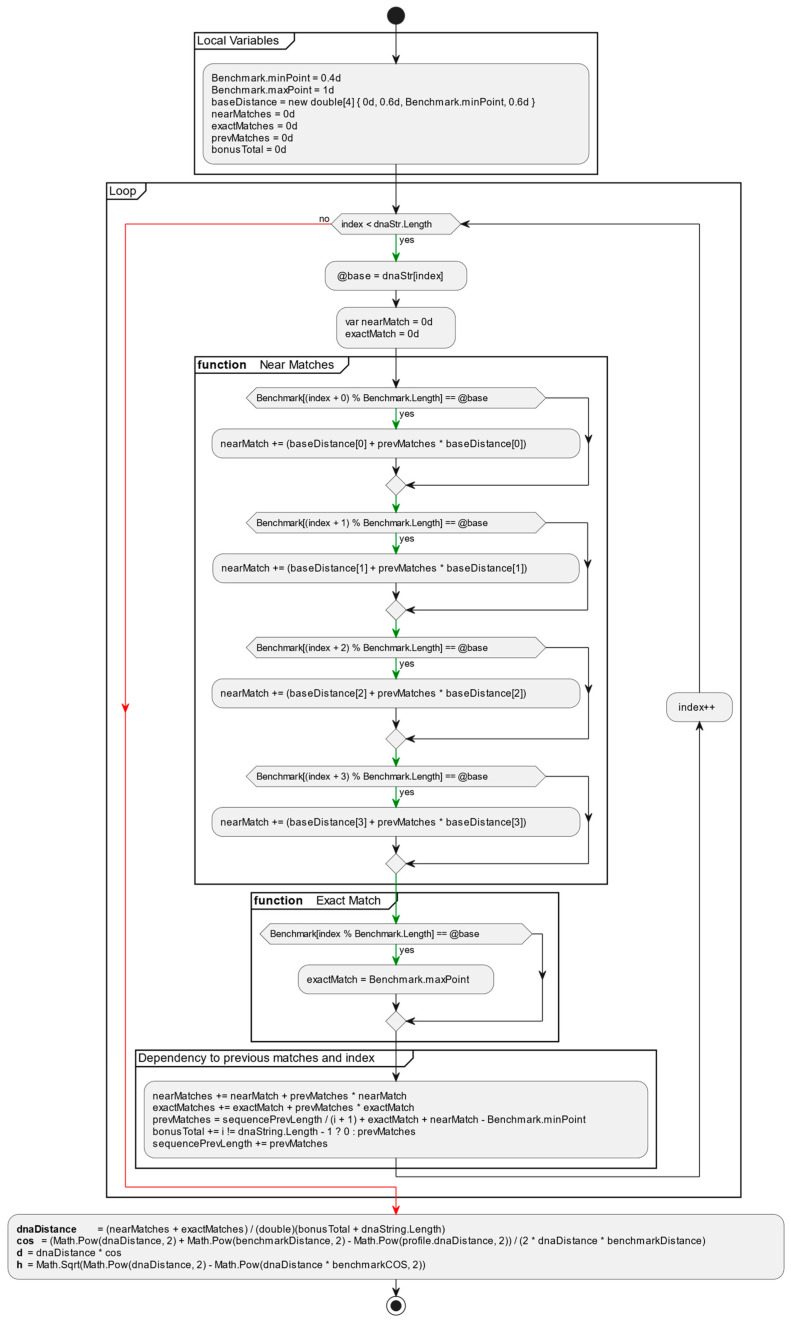
Algorithm for calculating a sequence profile against a benchmark.

The algorithm for aligning two DNA sequences using the proposed method unfolds in two distinct stages. Initially, the process involves generating a CAT profile for the input sequence by comparing it against a set of chosen benchmark sequences. This initial stage, despite being the most time-consuming part of the CAT method, is performed only once. For each selected benchmark sequence, a corresponding profile for the input sequence is calculated, resulting in the formation of a comprehensive CAT profile for the input sequence (Algorithm 1 and Figure 2).
**Algorithm 1:** Calculation of CAT profile for DNA sequence**Input:**DNA sequence as string (AGGTGCCGGT…….)**Output:**CAT profile: {C:{D,H}, A:{D,H}, T:{D,H}}**Processing Steps:**
**Step1:****Loop over sequence:** Count exact matches and near matches of the input DNA string. Consider when there were near or exact match with the previous comparison and sum bonuses given during the iteration. var nearMatch = benchmark.NearMatch(i, dnaString[i]); var exactMatch = benchmark.ExactMatch(i, dnaString[i]); nearMatches += nearMatch + prevMatches * nearMatch; exactMatches += exactMatch + prevMatches * exactMatch; prevMatches = sequencePrevLength/(i + 1) + exactMatch + nearMatch - Benchmark.minPoint; bonusTotal += i == dnaString.Length - 1 ? 0 : prevMatches; sequencePrevLength += (1 * prevMatches); **Step2:****For each benchmark:** dnaDistance = (nearMatches + exactMatches)/(bonusTotal + dnaString.Length); Calculate Cos(sequence benchmark distance, benchmark to benchmark distance) Calculate H(calculated benchmark cos) Calculate D(calculated benchmark cos)

This operation is performed once during the entry of the sequence into the database. The outcome is preserved as additional data associated with the sequence. This stage of the algorithm exhibits linear complexity, denoted as O(n).

The second stage involves a comparison against the previously calculated CAT profiles (Algorithm 2). This process is carried out iteratively when assessing the similarity between two or more sequences from the database. The DNA sequence alignment algorithm utilizing the CAT method maintains a constant complexity of O(21), making it highly efficient and easily implementable on computing machines. This characteristic significantly reduces the time required for searching large databases. To further enhance database speed, the algorithm can be parallelized by employing multiple threads for comparison against the computed CAT profiles. Additionally, it is feasible to set similarity limits for the search results, allowing the algorithm to consider not only exact matches but also similarities within defined limits.
**Algorithm 2:** Comparison of two DNA Sequence based on their CAT profiles**Input:**CAT profile1: {C:{D,H}, A:{D,H}, T:{D,H}}, CAT profile2: {C:{D,H}, A:{D,H}, T:{D,H}}**Output:**Comparison results in %**Processing Steps:**
**Step1:**resultC = Math.Sqrt(Math.Pow(x.c.D - y.c.D, 2) + Math.Pow(x.c.H - y.c.H, 2)); resultA = Math.Sqrt(Math.Pow(x.a.D - y.a.D, 2) + Math.Pow(x.a.H - y.a.H, 2)); resultT = Math.Sqrt(Math.Pow(x.t.D - y.t.D, 2) + Math.Pow(x.t.H - y.t.H, 2));**Step2:**Calculate result 1- (resultC + resultA + resultT)/3

For improvement in the accuracy of CAT in terms of precisely detecting the rate of similarities of sequences, we could change stage 2 of the algorithm to work with the areas of the triangles with vertex points from the corresponding benchmark of the CAT profile (D and h from Figure 1). We could use the Sutherland–Hodgman algorithm to identify an overlapping area of the compared profiles and then compute the area of the resulting convex polygon. The ratio of the resulting area to the minimal one from the two profiles’ area should give us theoretical optimal alignment. This will slow down stage 2 but keep its complexity as O(const). This enhancement will be explored in some of the upcoming research.

### 2.4. Implementation of the Proposed CAT Method for Sequence Alignment 

**Figure 3:**
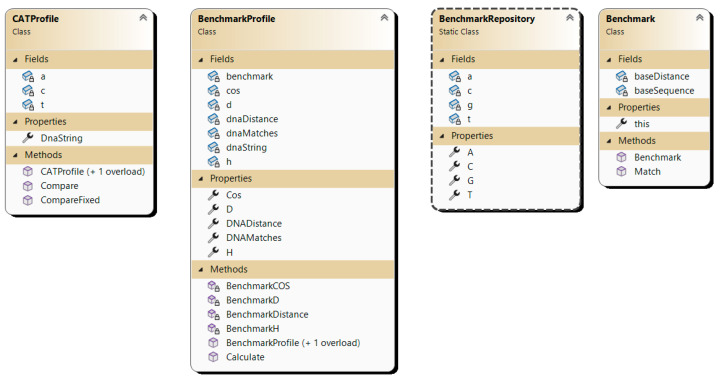
Class diagram of the program implementation using the CAT method for sequence alignment.

The aim of the experiments is to empirically assess the efficacy of the developed algorithm using the CAT method for DNA sequence alignment. To achieve this goal, a program implementation was developed using the C# programming language, and the class diagram is depicted in Figure 3.

- **Benchmark**: base class serving as an abstraction for representing benchmark sequences.
- **BenchmarkRepo**: repository containing predefined benchmark sequences.
- **BenchmarkProfile**: abstraction for plotting a DNA sequence against a benchmark sequence, calculating base parameters for the CAT comparison method such as Cos, D, and H.
- **CatProfile**: abstraction representing a DNA sequence with pre-calculated parameters for each benchmark sequence from the CAT method.

A sample code implementation of the proposed algorithm, protected by GNU General Public License v3.0, is available on GitHub: https://github.com/HristoS/CATSequenceAnalysis accessed on 30 January 2024.

## 3. Results

The newly introduced DNA sequence alignment method, CAT, leverages the trilateration technique and was empirically validated. It establishes three fixed benchmarks for implementing trilateration, thereby establishing a constant favorite sequence that does not vary with changes in the database records.

The key advantage of using constant benchmark sequences is that they are independent of the dataset and its size, allowing for sequence comparisons to be conducted at the initial stage of data uploading. This comparison produces metadata for each sequence, which significantly streamlines the process by eliminating the need for direct sequence comparisons during data retrieval; instead, the previously generated metadata is compared.

A detailed algorithm for DNA sequence alignment using the CAT method was designed. This includes an algorithm for generating a CAT profile using the predetermined benchmark sequences and a separate algorithm for comparing two sequences based on their respective CAT profiles. The steps for implementing this method, along with the required inputs and expected outputs, are clearly defined.

### 3.1. Collision Analysis

To assess the reliability of the CAT method, it is essential to explore the potential for collisions across the entire combination space, encompassing all permutations for a given sequence length. This prompts an inquiry into the uniqueness of the CAT profiles and the impact of accumulating statistical errors on the method’s reliability. To address this, all conceivable permutations of sequences with lengths 10, 11, 12, 13, and 15 were systematically generated (refer to Table 1, DNA length column). 

For each set, a sample of 1000 sequences was randomly selected and compared against the entire set. The count was carried out to determine how many of these sequences, after comparison with CAT, yielded a 100% match result (refer to Table 1, Average Collisions column). The collision rate is computed by dividing the total number of permutations (refer to Table 1, Total permutations column) for a sequence of a specific length by the instances of Average Collisions where CAT resulted in a 100% match (1).

From the table above, it can be observed that with the proposed implementation of CAT, there are no collisions found for the examined lengths. This makes CAT very reliable for finding exact sequence matches among sequences with equal length.

### 3.2. Performance Analysis 

An experiment was carried out to evaluate the comparison speed of CAT profiles. One hundred sequences of varying lengths (100, 1000, 10,000, and 50,000) were generated. CAT profiles were pre-calculated for these sequences. Subsequently, the CAT profiles, along with the Needleman–Wunsch and Knuth–Morris–Pratt algorithms, were compared against each sequence individually. The execution time for each comparison was recorded, and the outcomes are presented in Table 2.

**Figure 4:**
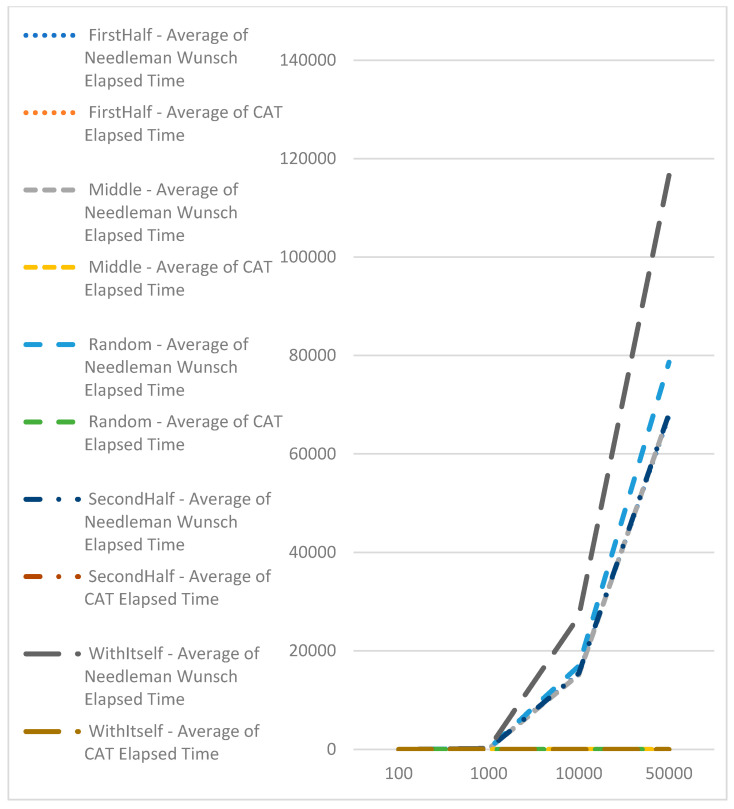
Performance comparison using CAT profiles and Needleman–Wunsch.

Table 2 indicates that the comparison times with CAT profiles are consistently close and do not vary based on the sequence length. In contrast, the comparison time using the Needleman–Wunsch algorithm exhibits exponential growth as the sequence length increases (refer to Figure 4).

**Figure 5:**
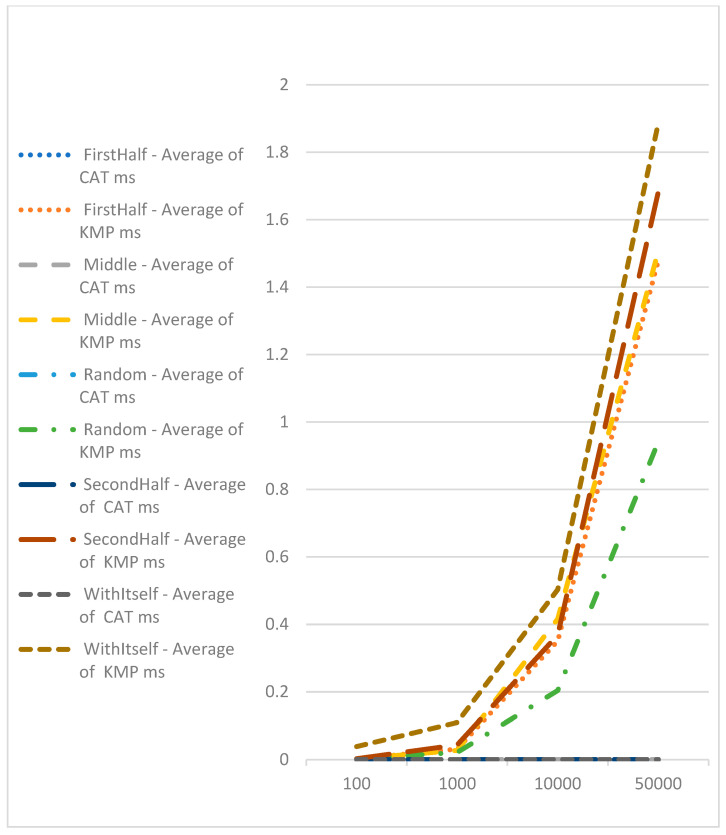
Performance comparison using CAT profiles and Knuth–Morris–Pratt.

Comparison with the Knuth–Morris–Pratt algorithm, which has a complexity of O(n), demonstrates the benefit of CAT-based algorithms for searching for exact matches of sequences (Figure 5). Here, we should point out that with the Knuth–Morris–Pratt algorithm, we could only identify the occurrence of subsequence in bigger or equal sequences. We cannot use this method for identifying similarities.

## 4. Discussion

The CAT methodology was validated via experimentation, introducing a novel approach that applies trilateration to generate a constant favorite sequence as a benchmark. This benchmark is unique in that it remains unchanged regardless of database changes, facilitating initial comparisons upon sequence database entry. These initial comparisons generate metadata for each sequence, eliminating the need for real-time sequence comparison during database queries, which is traditionally the most time-intensive step. 

The CAT method is structured to streamline the processing of input data, enabling the extraction and caching of CAT profiles against selected benchmark sequences and subsequently evaluating similarities based on these pre-generated profiles. This process ensures that the generation of CAT profiles occurs only once at the time of data uploading, allowing these profiles to serve as metadata. This innovative approach reduces search comparisons to a constant complexity of O(24), significantly enhancing search efficiency within extensive biological datasets. This efficiency positions the CAT method as an optimal preliminary step in more complex algorithms like FASTA, facilitating the organization of sequences into a hierarchical storage structure for optimized biological data storage and retrieval.

This new version of CAT builds on the trilateration method, improves the old one in the degree of collisions, and is just as fast. CAT profile creation occurs upon data entry into the database, allowing these profiles to act as metadata for the sequences. Dependency with a previous match and the closest neighbor is taken into consideration to increase the uniqueness of the CAT profile and to reduce possible collisions, i.e., two or more sequences having the same CAT profile. This makes the proposed algorithm suitable for finding the exact match of a concrete DNA sequence in a large set of DNA data.

Changes occur in the first stage of CAT profile calculation, and they do not affect the second stage, which is the actual alignment. With the new version, all experiments related to the examination of collisions were re-executed; no collisions were found, and some of the figures in the original article became redundant and were removed. In this paper, new results from the same experiments are exposed. Block schemes, pseudocodes, tables, and figures were updated according to the proposed new version and experimental results.

The experimental results demonstrate minor deviations in sequence alignment using the CAT method, which is deemed negligible given the substantial performance gains. Unlike the Needleman–Wunsch algorithm, whose execution time escalates with sequence length, the CAT method maintains consistent time efficiency across all various sequence lengths. This consistency underscores the capability of the CAT method to rapidly process alignments of extensive sequences, a task that would otherwise be time-prohibitive with exact algorithms. Various datasets have been tested to confirm the efficiency of the triplet-based CAT method, affirming its value in accelerating the alignment process while maintaining acceptable levels of accuracy. Experiments related to performance comparison with Needleman–Wunsch were re-executed with the new version of the algorithm to confirm that we have the same performance as the version presented in [9]. And we also added a performance comparison of the proposed DNA sequence alignment algorithm based on trilateration against Knuth–Morris–Pratt, which has a complexity of O(n) and is among the most commonly utilized for biological data searching. The results of the new experiments are represented in a table and in graphical formats for better understanding and as evidence of the advantages of the proposed modifications.

## 5. Conclusions

The approach of precomputing metadata and applying the trilateration principle provides a solution for the problem of slow alignment and similarity searching of biological data. Modification of the benchmark sequences and the way profiles are calculated and how they are compared results in the output of the comparison. This makes the approach adjustable to the desired level of accuracy. The experiments underscore the efficiency of the proposed algorithm and its potential to significantly speed up the process of DNA sequence alignment by leveraging the refined CAT profiles. The updated algorithm promises to be a valuable tool in bioinformatics, offering a faster and more reliable means for processing the vast and growing repositories of genetic data.

## Figures and Tables

**Table 1:** Collision comparison results.

DNA Length	Average Collisions	Total Permutations	Rate of Collision ‰ 0–1000
10	1	1,048,576	NaN
11	1	4,194,304	NaN
12	1	16,777,216	NaN
13	1	67,108,864	NaN
15	1	1,073,741,824	NaN

**Table 2:** Performance of CAT and Needleman–Wunsch comparisons.

**Average of CAT Elapsed Time**					
**DNA Length**	**First Half**	**Middle**	**Random**	**Second Half**	**With Itself**
100	0.0004025	0.000396	0.0004144	0.0003975	0.000254
1000	0.000466	0.0004495	0.0005036	0.0004555	0.0003
10,000	0.0007015	0.000644	0.0007408	0.0006945	0.00036
50,000	0.0003645	0.0003805	0.0003788	0.000356	0.000388
	**Average of Needleman–Wunsch Elapsed Time**				
	**First Half**	**Middle**	**Random**	**Second Half**	**With Itself**
100	0.810224833	0.8224725	0.875811143	0.807499499	1.59419
1000	137.5561462	137.9288827	148.2296519	136.8088905	238.639932
10,000	15,351.73013	15,130.27244	16,907.69611	15,416.20968	26,981.14435
50,000	67,806.26151	68,099.07336	78,652.58283	68,162.06354	116,611.3085
	**Average of Knuth–Morris–Pratt Elapsed Time**				
	**First Half**	**Middle**	**Random**	**Second Half**	**With Itself**
100	0.002494	0.002726	0.0018664	0.002865	0.038884
1000	0.0312275	0.0270915	0.0212584	0.043221	0.109476
10,000	0.350831	0.416332	0.2042432	0.368386	0.503456
50,000	1.4803245	1.507671	0.9423872	1.683394	1.88701

## Data Availability

Data are contained within the article. The permutations shown in Table 1 for the listed length are every time identical, and about the experiment itself, it could be easily repeated with the software implementation published in GitHub. Regarding the data in Table 2, it is generated by running the program published on GitHub, and on every run, we did get similar results. So, we would like to challenge the reader to play with the program and convince themselves that results we published are not coupled with the data at all and performance of CAT is approximately the same no matter of the data or its length. The software implementation is published at the following link: https://github.com/HristoS/CATSequenceAnalysis.
